# Knowledge Spillover and Emotional Motivation—A Study on the Willingness and Influencing Factors of Project-Based Learning

**DOI:** 10.3389/fpsyg.2022.795552

**Published:** 2022-05-17

**Authors:** Yanyan Ren

**Affiliations:** School of Foreign Languages and Literatures, Chongqing Normal University, Chongqing, China

**Keywords:** knowledge spillover, emotional motivation, willingness, influencing factors, project-based learning (PBL)

## Abstract

Project-based learning (PBL) is a task-based learning mode, which is of great significance to context-based curriculum education. With data collected through questionnaires and interviews, this article builds a binary logistic regression analysis model to explore the learners' willingness of PBL and its influencing factors. A total of 14 influencing factors are set in the research, with an innovative import of knowledge spillover and emotional motivation, to examine the impact on PBL willingness. Results show that eight factors, such as project-based learning experience, clarity of curriculum tasks, contextuality of curriculum content, and requirement for innovation of curriculum tools, have significant positive impacts on learners' willingness of PBL, while four factors, including the challenge of the curriculum project and tendency of learning objectives setting, have significant negative impacts. What's more, the grade of the learners and the challenge of the curriculum project have no significant impact. This article verifies that individual characteristics, knowledge spillover, and emotional motivation have different directions and degrees of impact on learners' willingness of PBL. Then, reasons are explored and implications proposed that these factors should be distinguished and paid sufficient attention to. Finally, suggestions are put forward on strengthening the contextuality of curriculum content, identifying the heterogeneity of learners, and expanding innovative tools to develop a PBL-based blended teaching model. In this way, the potential of PBL can be fully realized in maximizing learning achievements.

## Introduction

Problems, such as low student participation and poor knowledge spillover, are common in current tertiary education, which may be related to both teachers' curriculum design and students' resistance to traditional teaching methods. Under such circumstances, the traditional teaching model is in urgent need of reform and innovation.

In Bloom's Taxonomy, the cognitive process is divided into six stages: knowledge, comprehension, application, analysis, synthesis, and evaluation. The traditional classroom scenario is that teachers spend most of their time teaching knowledge and students are mainly involved in passive listening and note-taking, which represent a lower level of cognition (knowledge and comprehension). With the advancement of education concepts and the development of education models, teaching methods are also constantly changing. Project-based learning (PBL) emerges and gradually rises around the world under such background.

PBL has many advantages over other traditional teaching modes and can effectively improve learners' learning motivation and their learning achievements (Blumenfeld et al., 1991; Tabaku and Ecirli, 2014). It is generally believed that the adoption of PBL in college language teaching, especially in teaching which involves lots of application scenarios, such as business English teaching, can improve learners' satisfaction and meet their needs and desires in learning (Wang, 2012). However, as a novel teaching/learning model that is quite different from the traditional ones, PBL also imposes certain requirements for learners, which are generally considered to be affected by the learners' learning ability and motivation.

Knowledge spillover is a concept of externality. It was originally described in economic theory as a phenomenon that may have positive or negative impacts on things other than itself. It is considered to be an important source of economic growth (Saito and Gopinath, 2011). The achievement of economic performance depends not only on hard infrastructure (physical capital) but also increasingly on knowledge exchange and social infrastructure, including the availability and quality of human and social capital. In this regard, it has been verified that the spatial spillover effect of institutional quality exists (Sun et al., 2021b). Similarly, the change of teaching mode is also a kind of soft environment change, which has the characteristics of institutional change. In the learning process under PBL mode, knowledge spillover manifests itself as a positive externality. Learners gain project-based learning skills from skills already acquired and cooperation with their peers (Johnson and Johnson, 1999), to achieve positive learning effects under this mode.

In the field of production, studies on knowledge spillover argue that knowledge flows between paired sectors tend to be highly reciprocal, with no clear direction, and most sectors have relatively low concentrations of inflows and outflows. This suggests that most sectors become both knowledge providers and knowledge consumers, and develop together through mutual knowledge exchanges. However, as for individuals, the ability and tendency to accept new knowledge and new learning modes based on their learning skills is a manifestation of acknowledging the importance of prior knowledge from an individual's perspective, which will affect the individual's behavioral decision-making in the process of knowledge information transmission. This is also considered to be a manifestation of knowledge spillover. Although knowledge spillover is increasingly valued as a means to grasp innovation, little is known about its structural characteristics. In this article, basic language proficiency, attitude toward knowledge reserves, and other factors are introduced in the scope of the investigation to explore the mechanism of knowledge spillover in PBL, which is of great significance to the exploration of knowledge spillover in the field of language teaching.

Generally speaking, to enhance the attractiveness of a course and improve the teaching effects, teachers usually lay great emphasis on emotional factors, such as cultivating learners' interest and promoting their positive attitudes and evaluations. When learning brings happiness, satisfaction, and self-confidence to learners, they are more likely to be motivated to work harder, resulting in improved learning efficiency. However, these feelings belong to mood and are not sustainable (Perkins, 1991). Only by transforming mood into emotion can make learners to gain sustainable learning motivation, which will ultimately be beneficial to their learning effects. Emotional motivation has impact on both learners' willingness to learn and learning effects in the context of PBL. Therefore, we should extract key factors on the willingness of PBL to further improve project-based curriculum design and learning effects.

The learning willingness of PBL is closely related to the construction of the learner's knowledge system. It influences both teachers' curriculum design and learners' learning effects. By explaining knowledge spillover and emotional motivation in relation to the willingness of PBL, the main research focus of this study is as follows:

1. To analyze the influence mechanism of knowledge spillover and emotional motivation on learners' willingness of PBL.
2. To explore how to effectively improve learning effects under PBL mode.

## Literature Review

The mainstream knowledge spillover research focuses on knowledge spillover positioning, knowledge spillover aggregation, knowledge spillover institutional methods, knowledge spillover demography, entrepreneurial knowledge spillover, and so on. The nature of knowledge spillover is particularly important for controlling the impact of geographic location, and geographic proximity will affect knowledge transfer (Stolpe, 2002). Knowledge spillover theory based on innovative activities argues that geographic proximity is more important when tacit knowledge plays an important role in generating innovative activities. This theory reflects that knowledge transfer is subject to certain spatial constraints, and adjacent individuals are more likely to produce knowledge spillover effects.

In the institutional approach to knowledge spillover, the location of knowledge spillover as a type of economic cluster and the way knowledge spillover contributes to the development of regional economies should set the agenda for political practices (Varga, 2002). Countries with similar institutions may be more inclined to contact each other (Sun et al., 2021a). The institutional quality of a country may impact the institutional quality of a neighboring country, which subsequently affects their implemented energy regulations. Institutional spillover across countries may occur for a variety of reasons, that is, spatial institutional spillover.

Spillover effects can appear anywhere, such as in the flow of highly specialized professionals and when professionals apply knowledge to R&D services (human capital), thereby facilitating the formal and informal exchange of people and ideas and improving dissemination capabilities while improving operational efficiency (Acs, 2002; Delmar and Wennberg, 2010; Ellison et al., 2010). Based on this, Fernandes and Tang (2014) pointed out that in the knowledge spillover effect of universities and companies, geographic proximity influenced cooperation between companies and universities, and such cooperation influenced the capacity to undertake and generate innovation.

With the rapid development of information technology, it is necessary to use social networks as a promising tool to study the structure and evolution of intra- and interorganizational interaction and knowledge flow. Braunerhjelm et al. (2018) pointed out that there are inherently advanced knowledge flows among active individuals and that they have significant impacts on innovation. Knowledge spillover happens when knowledge is unintentionally shared among individuals, firms, and countries (Nicholas, 2013; Isaksson et al., 2016). The knowledge-sharing behavior of individuals has spillover effects, and it evolves with the change in information communication methods and informatization tools. The transfer of academic knowledge can be defined as a process based on understanding, information, and innovation (Parker and Zilberman, 1993). Knowledge spillover originates from knowledge reserves and is continuously updated and expanded through experiential learning. Knowledge exchange as a prerequisite for knowledge spillover is individually driven, so learners who are willing to share knowledge and build a scaffold for others usually achieve better learning outcomes. Under PBL, learners are organized in groups, and the knowledge of individuals diffuses both internally and externally, that is, both within themselves and among group members, which is a quite typical process. However, in current research, there is still a lack of exploration on knowledge spillover in PBL.

The theoretical basis of PBL is mainly constructivist learning theory and cognitive learning theory. Generally, it is believed that the main characteristics of PBL include real learning content, authentic assessment methods, teachers as facilitators, and clear educational goals. There is a correlation between the characteristics of PBL and students' learning motivation, and the impact of PBL on learning motivation is a major branch of language learning research (Wang, 2013). PBL should be more widely used in support of integrative approaches to sustainability, and it needs to become part of the routine of higher education institutions (Filho et al., 2016). Opinions from teachers toward PBL are also positive. It can promote the active participation of students, motivate them to learn, and help them to acquire various curricular skills (Basilotta Gomez-Pablos et al., 2017). The value of carrying out project-based learning lies in that core literacy and ability that could be promoted when learners were involved in asking questions and participating in in-depth exploration around challenging learning themes and projects (Hu, 2017). Moreover, the validity and effectiveness of the PBLCMM in identifying the potential for improvement suggest that educational institutions and teachers should apply a suitable approach to optimize the effectiveness of their PBL model (Al Mughrabi and Jaeger, 2018).

Business English is a course that learning should be rooted in various business situations and specific business communications. PBL, a teaching mode in which teaching activities are organized around a set of theme-related situations, is highly applicable in such courses, that is, business English learning should be project-oriented because of its distinguishing feature. Research has shown that in business English teaching, PBL has positive effects on learners for it is effective in stimulating learning motivation, enhancing language application ability, promoting cooperative learning, and cultivating comprehensive academic ability (Gao, 2010). The introduction of PBL into business English teaching could stimulate students' interests in learning, increase their classroom participation, cultivate their autonomous learning ability, and strengthen teacher–student and student–student interaction, and as a result, learning efficiency and teaching results could be effectively improved (Zhao, 2014). PBL is of practical significance for improving the effects of business English teaching. Proper integration of PBL in the teaching of business English enables students to learn knowledge by fulfilling complex and authentic business projects, thereby improving their problem-solving ability, autonomous learning ability, and lifelong learning ability (Feng, 2013). And the main teaching goal of project-based business English learning is to use knowledge and skills as the guiding principle and highlight the development of critical thinking ability (Liu and Pu, 2015).

Researchers agree on the positive role of PBL in language teaching. PBL in business English could drastically improve learners' autonomous learning consciousness and autonomous learning ability in the process of completing project tasks (Han, 2016). Under PBL mode, learners enjoy a more favorable experience in the information technology environment, cognition, society, and teaching, which in turn proves the positive impact of PBL on learning (Zhang, 2018). Skinner et al. (2015) explored learners' understanding of the purpose and value of learning groups in PBL, and their findings explain how students' epistemologies and perceptions of their learning contexts shape group behavior. However, PBL is not omnipotent. PBL was effective for students' knowledge, comprehension, and application-level achievements, but it had no significant effects on their self-regulation (Erdogan and Senemoglu, 2017).

Motivation is a critical factor in academic performance and active learning methods have positive impact on learners' motivation (Lopez Pena, 2018; Lopez-Fernandez et al., 2019). When learning English through task- and project-based learning, there are both challenges and opportunities for bilingual indigenous students. PBL is proved to be conducive to enhancing these students' speaking skills and facilitating their vocabulary recognition (Ricardo Rojas and Rueda Varon, 2019). When the teaching process is more focused (by content, time, and participants) on the task, better learning effects can be achieved. Therefore, changes in the PBL project structure should aim at solving wicked problems: introducing more diverse and smaller projects instead of one big project, teaching students how to manage information complexity and dynamic project requirements, and allowing students to participate in the selection and organization of learning teams (Kleczek et al., 2020).

Although a lot of research have been conducted around project-based learning, they mainly focus on its influence on enhancing learners' motivation and learning effects as well as its application strategies. As for the influencing factors of PBL, researches are scarce. Similarly, learners' willingness of PBL is also an area of rare research concern. In this article, a quantitative study is to be conducted to analyze learners' willingness of PLB and its influencing factors.

## Data, Variables and Research Model

### Data Source

Existing research have proved that PBL is of great significance in improving effectiveness of teaching. This article intends to explore university students' willingness of undertaking project-based learning in business English learning as well as its influencing factors from the dimensions of knowledge spillover and emotional motivation.

Data were collected through a combination of questionnaires and interviews. A total of 350 questionnaires were distributed to students at Chongqing University and Chongqing Normal University, and 328 were effectively recovered (recovery rate of 93.7%). After removing the unreasonable samples, a total of 320 valid questionnaires were obtained. To explore the potential appeal of PBL to learners, that is, the evaluation of knowledge spillover, students were interviewed about different PBL scenarios of business English during the data collection process. Thus, they have the opportunity to reflect and freely express their views toward PBL.

### Variables Description

Learners' emotions, thoughts, and behaviors have a huge impact on PBL (Frank et al., 2003). Goldman et al. (2020) identified project-based experiential learning as a defining element of quality improvement (QI) education, noting that ability, capacity, role clarity, and environment strongly influenced curriculum project success.

**Figure 1:**
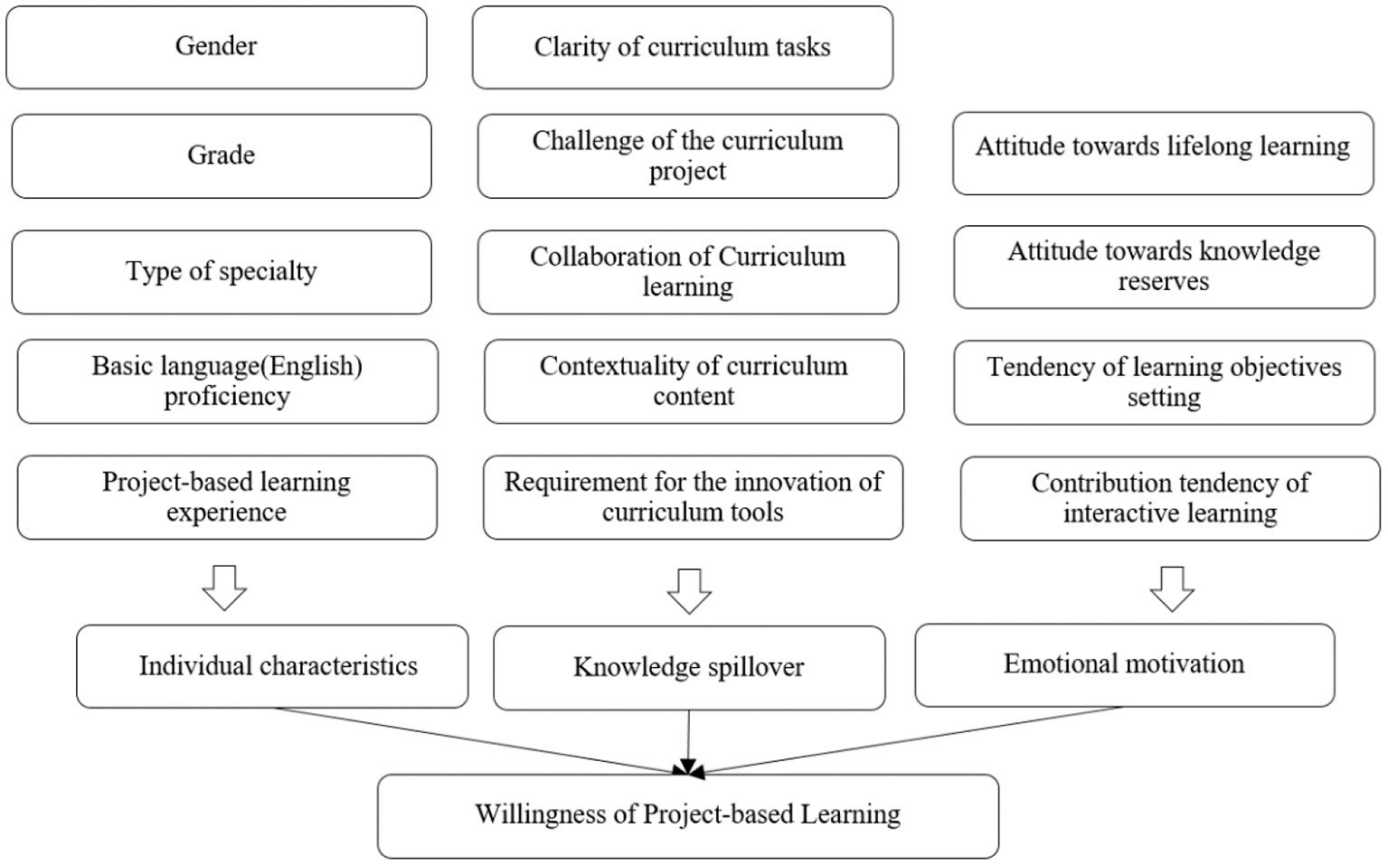
System of PBL willingness influencing factors.

In this article, the willingness of project-based learning is the explained variable. As for the influencing factors of PBL willingness, referring to previous literature, 14 explanatory variables are set in terms of learner individual characteristics ($\overset{5}{\underset{i=1}{\sum }}x_{i}$), knowledge spillover efficacy ($\overset{10}{\underset{i=6}{\sum }}x_{i}$), and emotional motivation ($\overset{14}{\underset{i=11}{\sum }}x_{i}$) (see Figure 1). Knowledge spillover efficacy is scaled by curriculum content factors. Emotional motivation is mainly manifested in the recognition of lifelong learning ability and the awareness of personal development.

Variables in statistics can be roughly divided into numerical variables and categorical variables. Numerical variables are numbers whose values can occupy certain columns, and these values are meant for operations, such as addition, subtraction, and averaging. Categorical variables, on the contrary, are meaningless for the above operation. In this article, all variables are categorical, and the explained variable of PBL willingness is binary. Each variable value is shown in Table 1.

**Table 1:** Basic statistics and variable assignment.

**Variable**	**Variable value**
Willingness of project-based learning	No (including the Don't know group) =0, Yes=1
Gender *x*_1_	Male=1, Female=2
Grade *x*_2_	Freshman=1, Sophomore=2, Junior=3, Senior=4, Postgraduate=5
Type of specialty *x*_3_	Arts=1, Sciences=2
Basic language (English) proficiency *x*_4_	Lower=1, General =2, Higher=3
Project-based learning experience *x*_5_	No=0, Yes=1
Clarity of curriculum tasks *x*_6_	No (including the Don't know group) =0, Yes=1
Challenge of the curriculum project *x*_7_	Lower=1, General=2, Higher=3
Collaboration of Curriculum learning *x*_8_	Lower=1, General=2, Higher=3
Contextuality of curriculum content *x*_9_	Lower=1, General=2, Higher=3
Requirement for the innovation of curriculum tools *x*_10_	Lower=1, General=2, Higher=3
Attitude toward lifelong learning *x*_11_	Totally deny=1, Comparatively deny=2, General=3, Comparatively accept =4, Totally accept=5
Attitude toward knowledge reserves *x*_12_	Passive=1, General=2, Active=3
Tendency of learning objectives setting *x*_13_	Highly unclear=1, Comparative unclear=2, General=3, Comparative clear=4, Highly clear=5
Contribution tendency of interactive learning *x*_14_	Highly reluctant=1, Comparative reluctant=2, General=3, Comparative enthusiastic=4, Highly enthusiastic=5

### Research Model

Willingness is a typical psychological tendency. The purpose of this study is to investigate this tendency in project-based learning and its influencing factors from the perspective of knowledge spillover and emotional motivation. Then, proper regulation can be implemented to improve learners' willingness of PBL.

Leaners' willingness of PBL, that is, learners' tendency toward PBL, is typically a problem within the domain of cognition. Since continuity data of effective time series is difficult to obtain for such problems, the probabilistic model is considered to be a relatively ideal estimation method (Hao, 2009).

Logistic regression is a generalized linear model, which can fundamentally solve the problems caused by the discontinuity of the dependent variable. It is often used in data mining, automatic disease diagnosis, economic prediction, and willingness selection research. There are two kinds of logistic regression models: binary and multinomial. For the binary logistic regression model, the dependent variable y has only two values: yes or no, recorded as 1 and 0, respectively.

Binary logistic regression is adopted in this article, based on which a multifactor analysis model of learners' willingness of PBL will be established. The functional relationship between the explained variable and the explanatory variables in this model is as follows:

Logistic Cumulative Distribution Function:

(1) $$\begin{array}{r} F\left(y\right)=\frac{e^{y}}{1+e^{y}} \end{array}$$

The ratio of the occurrence probability of a certain result to its non-occurrence probability is usually called the odds ratio, that is, under the action of p independent variables *x*_1_, *x*_2_, *x*_3_, ……*x*_*P*_, the probability of taking y as 1 is *p* = *P*(*y* = 1|X) and the probability of taking y as 0 is 1 − *p*.

Thus, the probability ratio of taking y as 1 or 0 is *p*/(1 − *p*). Then, in this research, the probability of learners' willingness of PBL is recorded as *p* = *P*(*y* = 1|X) and the probability of learners' unwillingness of PBL is 1-*p*. *p* and 1-p are shown in formulas (2) and (3):

(2) $$\begin{array}{r} p=P\left(y\text{=}1\;|\text{X}\right)=\frac{e^{f\left(x\right)}}{1+e^{f\left(x\right)}} \end{array}$$

(3) $$\begin{array}{r} 1-p=P\left(y=0|X\right)=\frac{1}{1+e^{f\left(x\right)}} \end{array}$$

After further transformation, the following model is formed:

(4) $$\begin{array}{r} \text{ln}{(}^{p}\text{​​}╱\text{​}{\text{​}}_{1-p}\;)=f\left(x\right)=\alpha +\overset{\text{n}}{\underset{i=1}{\sum }}\beta_{i}x_{i} \end{array}$$

In essence, the logistic regression model is a linear regression model established with $\text{ln}{(}^{p}\text{​​}╱\text{​}{\text{​}}_{1-p}\;)$ and natural variables. The value range of $\text{ln}{(}^{p}\text{​​}╱\text{​}{\text{​}}_{1-p}\;)$ is (−∞, +∞). Then, the value of the independent variables *x*_1_, *x*_2_, *x*_3_, ……*x*_*i*_ can be anything in the range.

*x*_*i*_ refers to the explanatory variable. α represents a constant term, which is the natural logarithm of the ratio of occurrence probability of *y* = 1 and *y* = 0 when there is no independent variable, that is, the value of *x*_1_, *x*_2_, *x*_3_, ……*x*_*i*_ is 0. β_*i*_ refers to the coefficient of the explanatory variable, which is the logarithm of odds (*y* = 1) when an independent variable *X* change, that is, *X* = 1 or *X* = 0.

## Results Analysis of the Regression Model

Results indicate that the majority of respondents claimed that they are willing to participate in PBL curriculums, although they might not have real project-based learning experience yet. Descriptive statistics of the variables are shown in Table 2.

**Table 2:** Descriptive statistics of the variables.

**Variable**	***N***	**Min**	**Max**	**Mean**
Willingness of project-based learning	320	0	1	0.71
Gender	320	1	2	1.53
Grade	320	1	5	2.90
Type of specialty	320	1	2	1.45
Basic language (English) proficiency	320	1	3	2.01
Project-based learning experience	320	0	1	0.60
Clarity of curriculum tasks	320	0	1	0.65
Challenge of the curriculum project	320	1	3	2.19
Collaboration of Curriculum learning	320	1	3	2.41
Contextuality of curriculum content	320	1	3	2.34
Requirement for the innovation of curriculum tools	320	1	3	1.99
Attitude toward lifelong learning	320	3	5	4.28
Attitude toward knowledge reserves	320	1	3	2.39
Tendency of learning objectives setting	320	1	5	3.34
Contribution tendency of interactive learning	320	2	5	4.01

**Figure 2:**
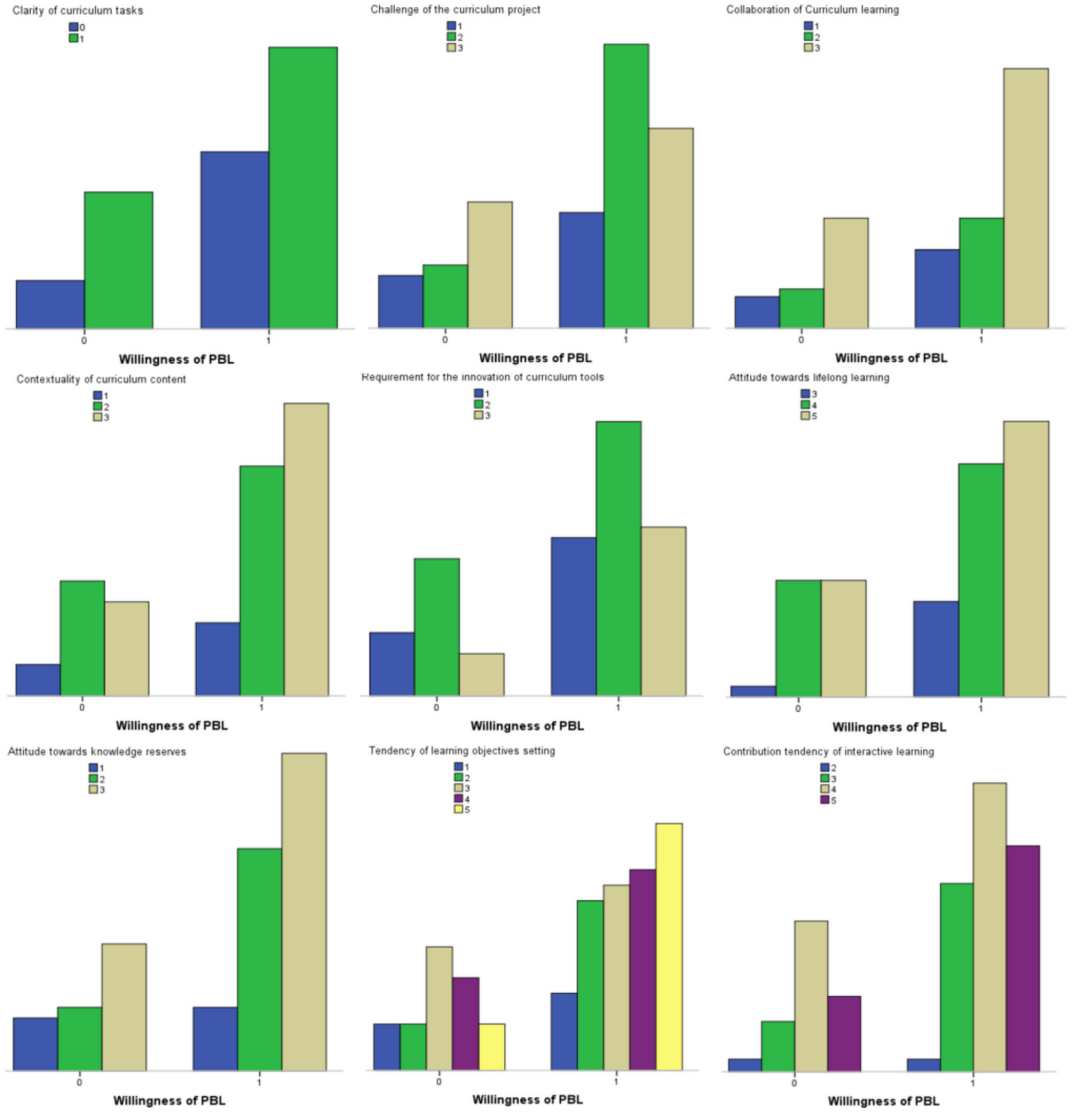
Cross-analysis diagram of influencing factors of knowledge spillover.

For the basic statistical description, this article conducts a cross-analysis on variables of knowledge spillover and emotional motivation (see Figure 2). It shows that willingness of PBL has a certain relationship with knowledge spillover and emotional motivation factors.

**Figure 3:**
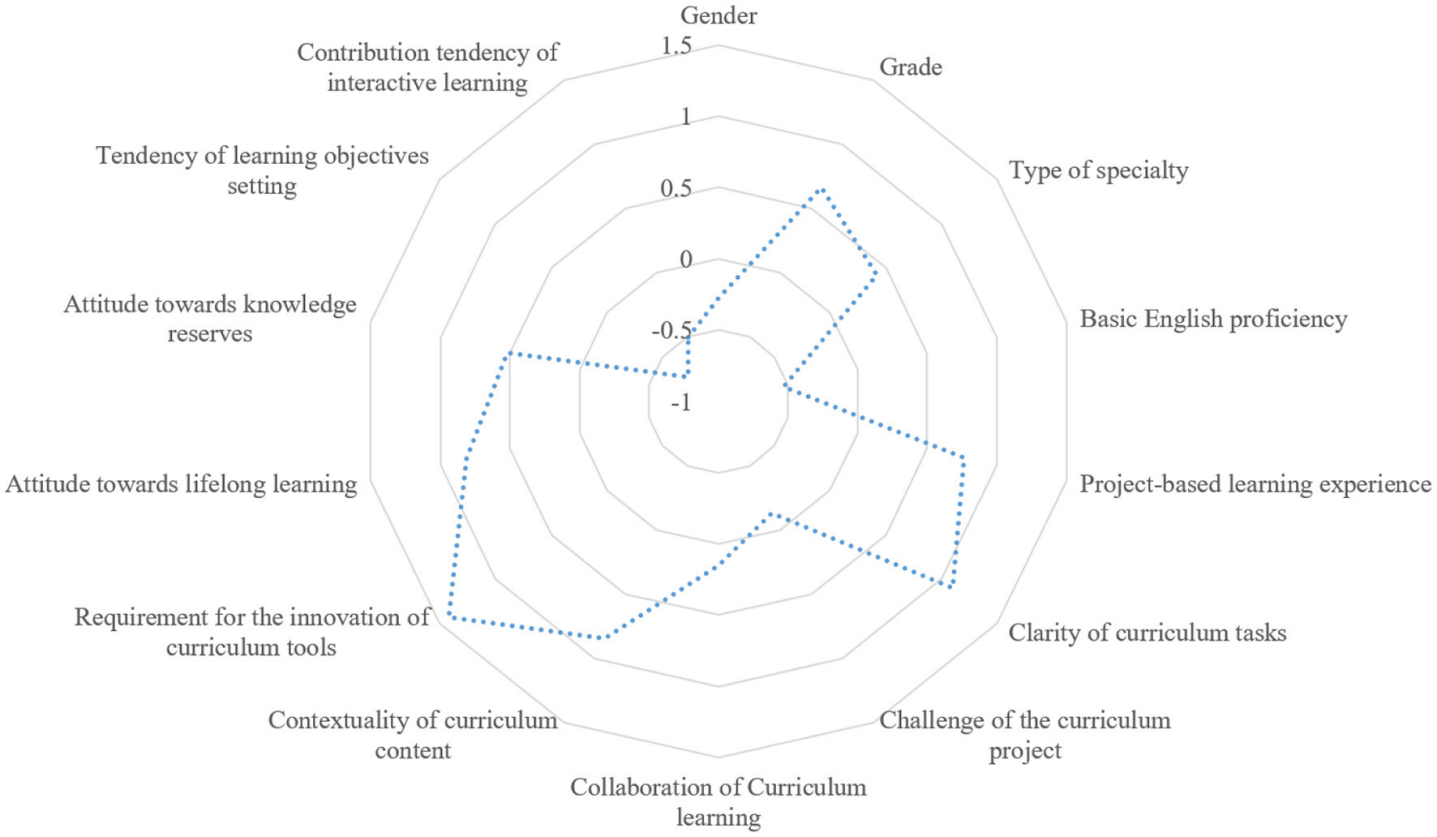
State distribution of regression results of influencing factors.

Results of the model estimation manifest that clarity of curriculum tasks, contextuality of curriculum content, and requirement for the innovation of curriculum tools have a comparatively greater impact on learners' willingness of PBL (see Figure 3).

With the above-mentioned binary logistic regression model, SPSS19.0 is used to examine the willingness parameters of PBL. Results are shown in Table 3. Some of the influencing factors show positive impacts on learners' willingness of PBL, some show negative impacts, and others have no impact at all. Factors that have significant positive impacts are a type of specialty, project-based learning experience, clarity of curriculum tasks, collaboration of curriculum learning, contextuality of curriculum content, requirements for the innovation of curriculum tools, attitude toward lifelong learning and attitudes toward knowledge reserves, and factors including gender, basic language proficiency, the tendency of learning objectives setting, and contribution tendency of interactive learning have significant negative effects. However, two factors namely grade and the challenge of the curriculum project show no statistically significant effect on learners' willingness of PBL.

**Table 3:** Statistics and variable assignment.

**Covariates**	**B**	**S.E**,	**Wald**	**df**	**Sig**.	**Exp (B)**
Gender	−0.281	0.152	3.418	1	0.027	0.755
Grade	0.665	0.436	2.326	1	0.927	1.944
Type of specialty	0.419	0.133	9.925	1	0.019	1.520
Basic language (English) proficiency	−0.536	0.209	6.577	1	0.043	0.585
Project-based learning experience	0.764	0.351	4.738	1	0.042	2.147
Clarity of curriculum tasks	1.103	0.467	5.578	1	0.017	3.013
Challenge of the curriculum project	−0.133	0.79	0.028	1	0.584	0.875
Collaboration of Curriculum learning	0.151	0.114	1.754	1	0.001	1.163
Contextuality of curriculum content	0.847	0.367	5.326	1	0.000	2.333
Requirement for the innovation of curriculum tools	1.423	0.652	4.763	1	0.029	4.150
Attitude toward lifelong learning	0.81	0.56	2.092	1	0.018	2.248
Attitude toward knowledge reserves	0.518	0.343	2.281	1	0.033	1.679
Tendency of learning objectives setting	−0.726	0.431	2.837	1	0.012	0.484
Contribution tendency of interactive learning	−0.503	0.321	2.455	1	0.041	0.605

Among our survey participants, 72.23% of Arts learners have the willingness of PBL and 66.67% of Science learners report such willingness, which demonstrates that type of specialty, has positive impact on learners' willingness of PBL. This might be ascribed to the fact that the knowledge attribute of the art courses is more discrete than that of the science courses; consequently, it is easier for learners specialized in such fields to understand the concept and mode of PBL. Learners with the project-based learning experience, which is supposed to have the effect to improve learning effectiveness, are more inclined to accept this learning mode. Such a result confirms and consolidates previous research findings (Zhang and Hu, 2019). Interestingly, in our survey, the proportion of male students who are willing to accept PBL is 78.95%, while only 61.9% of female students are willing to accept it. Compared with female students, male students' willingness of PBL is significantly higher than female students; that is to say, gender has negative impact on the willingness of PBL as shown in Table 3. This may have something to do with the fact that male students are perceived to be more eager to try new and different learning methods after entering university.

The reason why clarity of curriculum tasks has a positive effect may be that learners are more willing to learn under a clearly oriented teaching mode, especially when learning new knowledge in the university. Knowledge spillover in this study refers specifically to the learners' ability and tendency to accept new knowledge and new learning modes based on their learning skills. The analysis results show that the higher the collaboration of curriculum learning, the stronger the contextuality of curriculum content, and the higher the innovation of the curriculum tools, the easier it is to enhance learners' willingness of PBL. These three covariates are important components of PBL and, at the same time, they are the results of learners' extension of their learning skills. This further verifies that there is a certain interaction mechanism between the content design of PBL and learner skills.

It is noteworthy that the learner's basic language (English) proficiency shows negative impacts on learners' willingness of PBL, which may be because lower level learners expect, through fulfilling tasks in PBL, to achieve inner emotional promotion, including promoting their interest in English learning. However, learners of higher basic language (English) proficiency, with strong learning ability and a solid foundation of English, do not rely so much on the teaching mode.

Attitudes toward lifelong learning and attitudes toward knowledge reserves positively affect learners' willingness of PBL, which may be attributed to the fact that learners endorse the positive impact of emotional motivation on learning effects. This further suggests that in the process of project-based learning, more attention should be paid to learner emotions to improve learning efficiency. However, both the tendency of learning objectives setting and the contribution tendency of interactive learning have negative impacts on PBL. Learners with lower evaluation of self-learning settings are more likely to accept PBL than those with higher evaluation. This phenomenon might be ascribed to that when learners lack objective awareness, they would reshape the learning process and learning objectives with the help of external forces. These external forces can be obtained in the typical process of project-based learning and the curriculum design based on this learning mode. Similarly, learners with a lower contribution tendency of interactive learning are of higher willingness to accept PBL. Reasons for this might be that these learners are more likely to “hitchhike” under the PBL environment, which, in turn, serves as a reminder for teachers when developing learning projects. It is essential to differentiate and pay due attention to the individual differences of learners instead of contenting on learners' fulfilling the project in learning groups as a whole.

Among the factors examined by our research model, grade and challenge of the curriculum project have no significant impact on learners' willingness of PBL. It is found in the survey that the learning of university students, regardless of grades, is not linear but segmented, and there is no special requirement for any specified learning mode. That explained why learners of different grades show no significant difference in terms of the willingness of PBL. The challenge of the curriculum project is not a criterion for learners to opt for a certain teaching mode. They make choices according to their intrinsic motivation rather than the explicit representation of a teaching mode. Thus, it is not striking that challenge of the curriculum project does not influence learners' willingness of PBL significantly.

## Conclusion and Suggestions

Project-based learning (PBL) is a task-based learning model, which is of great significance to contextualized curriculum education. This study takes university students as research objects to explore the influencing factors of learners' willingness of PBL. Findings are that factors of knowledge spillover and emotional motivation have significant impacts on learners' willingness of PBL. First, type of specialty, project-based learning experience, clarity of curriculum tasks, collaboration of curriculum learning, contextuality of curriculum content, requirement for the innovation of curriculum tools, attitude toward lifelong learning, attitude toward knowledge reserves have significant positive impacts on learners' willingness of PBL. Second, basic language (English) proficiency, the tendency of learning objectives setting, and the contribution tendency of interactive learning have significant negative impacts on learners' willingness of PBL. Third, the grade of learners and the challenge of the curriculum project have no significant impact on learners' willingness of PBL.

However, it should be acknowledged that this study is not without limitations. First, the sample is not large enough to include students from all majors. However, due to the differences in professional attributes, students' willingness toward a certain teaching mode might not be the same. Therefore, further research should be done to examine a wider range of samples to include students from all professional attributes and cultural backgrounds. Second, willingness is a typical psychological disposition, and wider sampling studies can provide a more in-depth discussion of the general applicability of PBL. Despite these limitations, this study remains of great value. It provides a theoretical explanation for the influence of knowledge spillover and emotional motivation on PBL learning willingness, and the findings can guide teachers to take effective measures in the teaching process to achieve better teaching results.

**Figure 4:**
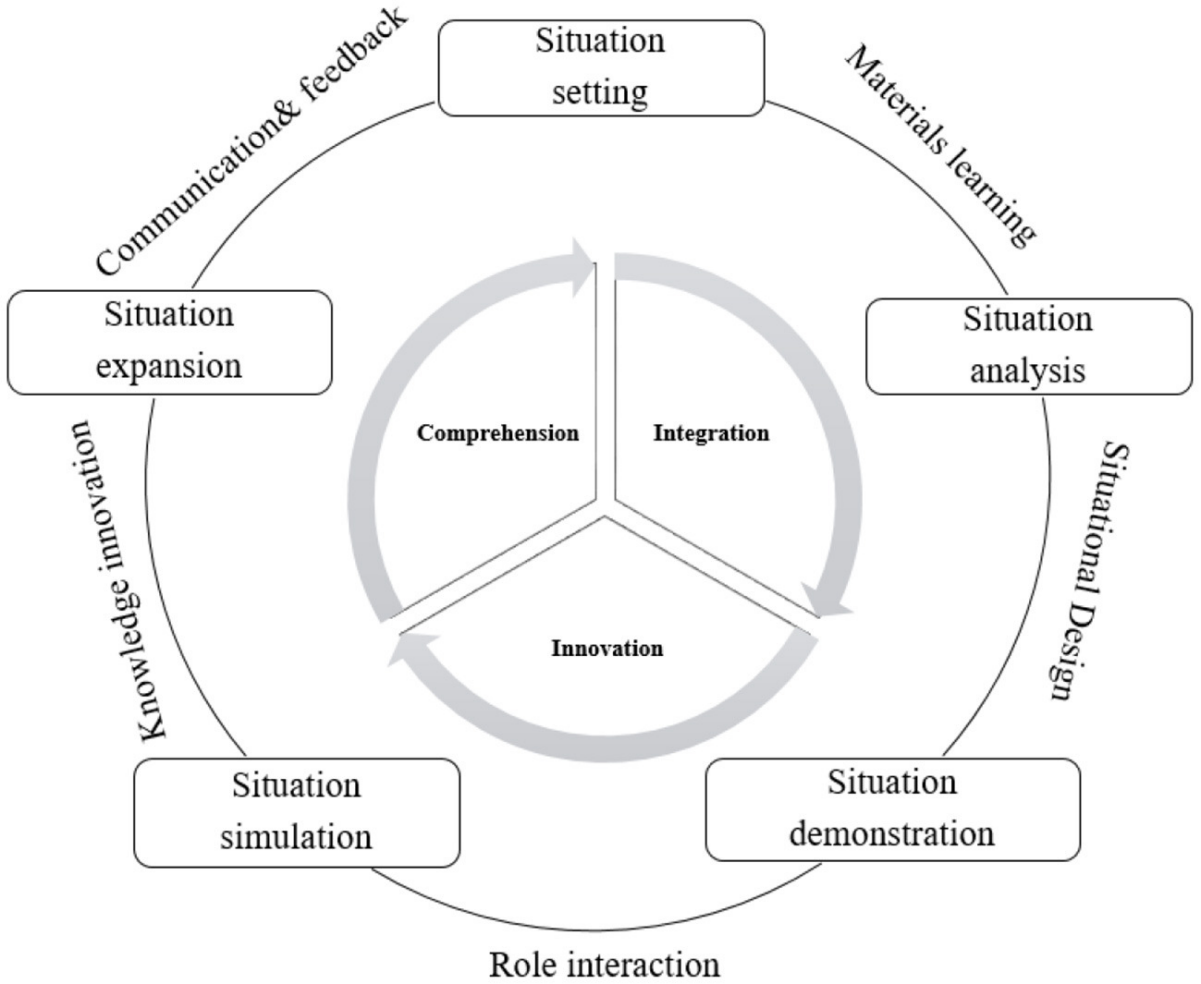
Route for the construction of educational situations.

As it has been proved, PBL is an efficient teaching mode that is conducive to facilitating the comprehensive development of learners. To obtain maximum learning effects under PBL mode, the following suggestions are proposed:

(1) Strengthen the contextuality of curriculum content: It is agreed that PBL has the characteristic of clear tasks in the form of projects. Teaching activities are organized around these tasks, with each task representing a specific knowledge point. In teaching activities under PBL, situations should be constructed when designing curriculum and projects, so that learning effects can be improved through situation setting, situation analysis, situation demonstration, situation simulation, and situation expansion (see Figure 4). When learners are involved in situational curriculum projects with specific goals, their understanding of knowledge will be facilitated and their comprehensive ability to use the language will be promoted. The key point of situation construction is to improve the simulation path of situations. In curriculum design, dramatic means, such as role-play, can be adopted to enhance learners' sense of participation.

(2) Identify the heterogeneity of learners: PBL begins with task-based teaching activities, namely, projects; however, it is found in the research that “hitchhiking” is common among some learners. Therefore, due attention should be paid to the assessment of learners' contribution rate in both curriculum design and project implementation. Learners' participation abilities and tendencies should be differentiated and properly guided to ensure that their self-assessment is in accordance with the curriculum goals.

**Figure 5:**
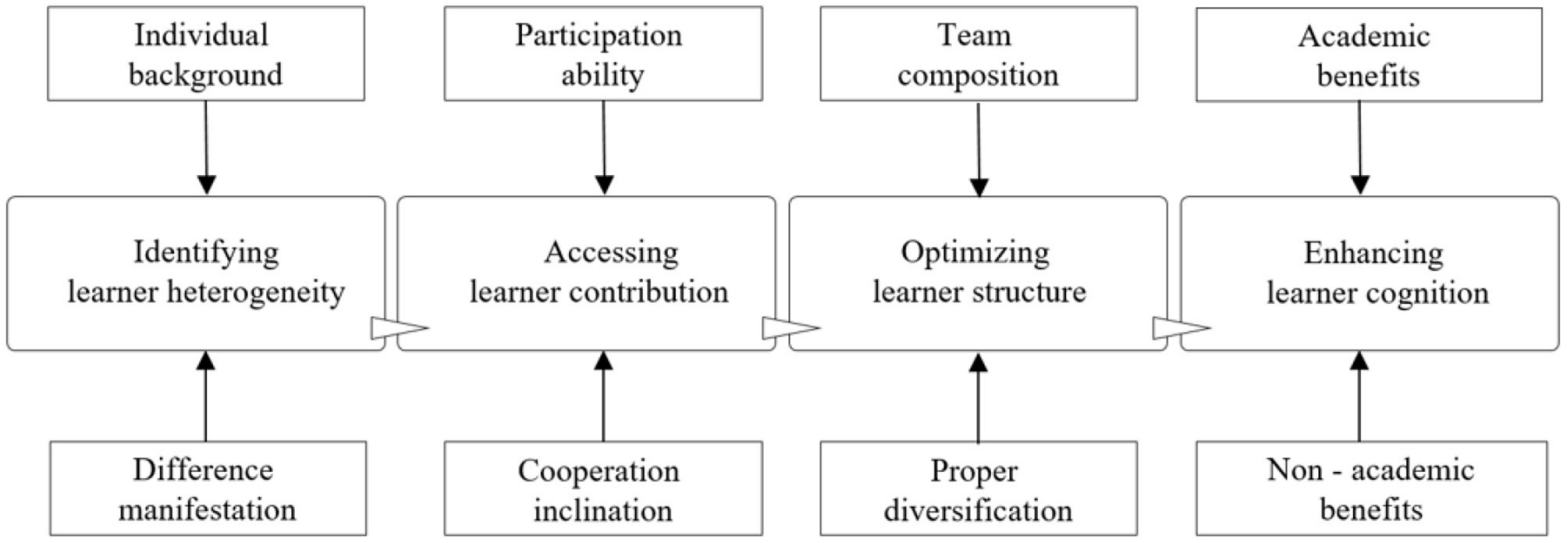
Process of PBL benefits.

Research shows that the main challenges for learners engaged in PBL are time management and teamwork, whereas the main benefits from engaging in PBL are both academic and non-academic benefits. Non-academic benefits include improving communication and negotiation skills, time management, creativity, and critical thinking ability, which are exactly the challenges learners are confronted with. In PBL, passive learners should be paired with active learners to improve the overall gains from project participation. In other words, based on screening and differentiating, optimizing the structure of learners is of substantial significance for improving the learning efficiency of PBL. In the process of project participation, proper allocation of learners of different genders and majors and appropriate size of project learning groups can enhance the cognitive level of PBL teams as a whole. The process of PBL benefits is shown in Figure 5.

(3) Expand innovative tools and develop a blended teaching mode. In PBL, innovative tools are often demanded by learners to enhance the learning experience. Therefore, it is suggested to create a learning environment of greater practical significance by utilizing information networks and terminal products in the teaching process. PBL should be implemented in the context of blended teaching mode, making full use of online and offline resources. The establishment of a PBL-based blended teaching mode requires attention to the processes and conditions of different types of learning. While advocating for learners to take the initiative, the project-oriented learning experience should be gradually and progressively accumulated, and external technical support for learning and the internal drive of project design should be strengthened. The blended teaching mode is conducive to stimulating learner initiative and improving teaching supervision and evaluation, which can greatly enrich the construction of project situations and expand interactive exchanges.

However, the ultimate goal of applying project-based blended teaching mode is neither to utilize various online platforms and build digital teaching resources nor to carry out fancy teaching activities but to effectively deepen the depth and expand the width of learning for learners. This is also the direction for further research on PBL.

## Data Availability Statement

The original contributions presented in the study are included in the article/supplementary material, further inquiries can be directed to the corresponding authors.

## Ethics Statement

The studies involving human participants were reviewed and approved by Chongqing Normal University. The participants provided their written informed consent to participate in this study.

## Author Contributions

YR designed the research framework, analyzed the data, and wrote and edited the manuscript.

## Funding

This research was supported by Chongqing Tertiary Education Teaching Reform Research Project (Fund Number: 183046).

## Conflict of Interest

The author declares that the research was conducted in the absence of any commercial or financial relationships that could be construed as a potential conflict of interest.

## Publisher's Note

All claims expressed in this article are solely those of the authors and do not necessarily represent those of their affiliated organizations, or those of the publisher, the editors and the reviewers. Any product that may be evaluated in this article, or claim that may be made by its manufacturer, is not guaranteed or endorsed by the publisher.
